# A preliminary investigation of presenteeism and cognitive preferences among head nurses: a cross-sectional study

**DOI:** 10.1186/s12912-023-01498-0

**Published:** 2023-09-27

**Authors:** Wenzhen Li, Geyan Shan, Shengnan Wang, Hongxia Wang, Wei Wang, Yongxin Li

**Affiliations:** 1 Institute of Psychology and Behavior, Henan University, Kaifeng, Henan Province China; 2 Business School, Henan University, Kaifeng, China; 3 Institute of International Education, Henan University of Animal Husbandry and Economy, Zhengzhou, China; 4 Department of Outpatient, Henan Province Hospital of TCM, Zhengzhou, China

**Keywords:** Presenteeism, Cognitive preference, Head nurse, Cross-sectional survey

## Abstract

**Background:**

Individual health is essential for productivity at work. However, presenteeism, which is defined as attending work while ill, is common. Nursing is a profession with a high incidence of presenteeism, leading to diverse negative outcomes. Considering the unique and significant role of head nurses and the influence of cognitive factors on presenteeism, the current study aimed to investigate the incidence of presenteeism among head nurses, their cognitive preference towards presenteeism, and the association between the two.

**Methods:**

This preliminary investigation was a cross-sectional study conducted from July to August 2022. Participants were 233 head nurses recruited via convenience sampling from six hospitals located in Zhengzhou, Henan Province, China. The Nurse Presenteeism Questionniare (NPQ) and an original cognitive preference questionnaire were used to measure head nurses’ experience of presenteeism and cognitive preference towards presenteeism. Descriptive statistics and sample t-tests were performed for data analysis.

**Results:**

In the past six months, 96.6% of the head nurses exhibited signs of presenteeism. The specific symptoms were discomfort in the lower back, dizziness or headache, cold (e.g., stuffy nose or cough), abdominal pain (including menstrual pain), and whole-body fatigue or discomfort. 95.7% of head nurses’ anticipation preference toward presenteeism inclined to rest at home; additionally, more than 80% of the head nurses considered presenteeism detrimental to both individuals and organizations. Further, 63.9% of the head nurses were inclined toward conduct discouragement in the face of subordinates’ presenteeism. There was no significant difference in presenteeism between head nurses with various anticipation preferences (*p* > 0.05) and benefit preferences (*p* > 0.05). However, the differences in presenteeism among head nurses with various management preferences were significant (*t* = 2.60, *p* = 0.01). Specifically, head nurses who favored encouraging subordinate presenteeism had higher presenteeism scores compared to those who discouraged it.

**Conclusions:**

Presenteeism among head nurses remains a universal workplace phenomenon. There was inconsistency among head nurses’ anticipation preferences, benefit preferences, and presenteeism. However, there was consistency between head nurses’ management preferences and presenteeism.

## Introduction

Nurses have been vital caregivers contributing in crucial and diverse ways to health systems, especially during the COVID-19 pandemic [1]. Considering the essential role of nurses in health systems, protecting nurses’ professional health and valuing the nursing profession are effective approaches to strengthening health systems [2]. However, presenteeism, defined as “the phenomenon of people, despite complaints and ill health that should prompt rest and absence from work, still turning up at their jobs” [3], poses a risk to nurses and health systems [4, 5]. Presenteeism decreases nurses’ physical and mental health [6] while increasing individual burnout [7] and social financial burden [8]. Furthermore, presenteeism among nurses is globally prevalent [9], with the percentage of presenteeism varying from 30% to more than 90% [10]. Specifically, the incidence of presenteeism among Spanish medical staff is 53% [11]; presenteeism is reported in 91.4% of Portuguese nurses [12]; its prevalence among Chinese nurses is 94.25% [13]; among Swedish senior nurses, registered nurses, and assistant nurses, it is approximately 50% [3].

Nevertheless, although head nurses are a particular and essential subgroup among nurses, their presenteeism has been ignored. It is known that head nurses have unique roles compared with general nurses and they are important to nursing departments [14, 15]. The occupational roles of general nurses and head nurses are widely different; consequently, the intrinsic mechanism of presenteeism among head nurses is complex and differs from that of general nurses. Additionally, as the direct leaders, head nurses are a model for general nurses [14, 16, 17], which positively affects their subordinates’ presenteeism through a “trickle-down effect.” Considering head nurses’ presenteeism and its vital impact on subordinates, this study aimed to investigate the incidence of presenteeism among head nurses.

It is essential to exclusively explore predictors of head nurses’ presenteeism since head nurses impact subordinate nurses. According to the research agenda regarding presenteeism, Johns [18] has claimed that individual factors influence the choice between a sick leave and presenteeism. Specifically, personal factors, such as individual high affective commitment, work engagement, and job satisfaction, promote employees’ tendency to go to work while ill [18, 19]. Additionally, recent studies have latently explored how individual cognitive factors, namely, individual subjective perceptions, impact the occurrence of presenteeism, suggesting that employees’ perceptions of the legitimacy of presenteeism and institutional pressure significantly affects individual presenteeism [20, 21]. However, while researchers have focused on the impact of individual underlying perceptions on presenteeism, they have examined perceptions that are influenced by organizational environment, rather than cognition based on individual attitudes and value. However, individual internal cognition is a non-negligible variable influencing individual behaviors, since the brain receives external information and transforms it into internal psychological activities, which further dominate behaviors [22]. Accordingly, based on the definition of cognition [23], we introduced head nurses’ cognitive preference towards presenteeism as individual intrinsic cognition, that is, head nurses’ preference towards presenteeism, including the anticipation of possible presenteeism, the interpretation of current presenteeism, and the evaluation of past presenteeism. Furthermore, we developed the Cognitive Preference Questionnaire to measure head nurses’ cognitive preference towards presenteeism with the guidance of experimental vignette methodology and an expert evaluation method. This study aimed to preliminarily inspect head nurses’ cognitive preference towards presenteeism via a scenario questionnaire. Furthermore, this study aimed to explore the relationship between head nurses’ cognitive preference regarding presenteeism and the actual occurrence of presenteeism among head nurses.

We aimed to contribute to the presenteeism literature in two ways. First, our study introduces cognitive preference toward presenteeism and contributes to the influence of cognitive factors on presenteeism, thereby advancing the understanding of the boundary conditions of presenteeism. Second, we focused on the occupational characteristics and essential role of head nurses to explore the underlying mechanisms of presenteeism among nurses.

## Background

### Presenteeism among head nurses

Head nurse is a demanding, multifaceted, and complicated position which serves a unique role in nursing departments [14]. Specifically, head nurses engage in nursing care within their departments and act as caregivers. Additionally, head nurses undertake responsibilities as supervisors and act as leaders. Compared with general nurses, head nurses have extra roles specific to the job, serving the dual roles of caregiver and leader. Accordingly, presenteeism among head nurses is likely more prevalent than among general nurses, since previous research has demonstrated that professional self-identity affects nurses’ presenteeism [5]. Specifically, their personal sense of their role as a nurse influences subsequent presenteeism decisions by guiding their evaluations of illness [24], health locus of control [24], and individual responsibility and commitment [25]. From the caregiving perspective, head nurses’ professional self-identity promotes the incidence of presenteeism. Additionally, from the leadership perspective, head nurses’ individual identity positively impacts presenteeism. Cullen and McLaughlin [26] have discussed presenteeism in the Irish hospitality industry and have found that self-identified leaders prefer to encourage presenteeism and consider it a valuable behavior. According to the occupational roles and characteristics of general nurses and head nurses, the underling mechanism of presenteeism among head nurses is complicated and differs from that among general nurses.

Furthermore, head nurses’ presenteeism retains enormous influence in nursing departments, that is, significantly impacting subordinates’ presenteeism. Head nurses, as the direct leaders, typically model subordinates’ work behavior, including presenteeism [14, 17]. More specifically, the choices made by head nurses while ill, consisting of either sick absences or presenteeism, may impact subordinate nurses through a “trickle-down effect,” resulting in general nurses making similar choices when sick. Shan et al. [14] have claimed that presenteeism among head nurses positively affects their subordinate presenteeism, suggesting that subordinate nurses are likely to model head nurses’ presenteeism. Additionally, head nurses are the dominate shaper of organizational culture on presenteeism [16]. Ruhle and Süß [20] have stated that employees adapted their presenteeism based on their perception of organizational climate, which is formed by the legitimacy of presenteeism from supervisors. Therefore, head nurses’ presenteeism is an essential signal of subordinate nurses’ presenteeism.

Therefore, considering the particular and extra occupational roles of head nurses and the remarkable impact of head nurses’ presenteeism within nursing departments, the first objective of this study is proposed:

Objective 1: investigate the incidence of presenteeism among head nurses.

### Head nurses’ cognitive preference toward presenteeism

Individual factors are the significant antecedents of presenteeism among head nurses. Considering the extensive influence of head nurses’ presenteeism on subordinates in nursing departments, the exploration of predictors is essential [14]. Johns [18] has postulated that two main dimensions affect presenteeism: individual factors and situational factors. Individual factors refer to between-individual variables, such as work attitude, personality, gender, perceived work stress and among others. Existing research has shown that nurses’ attitudes toward work tasks and the organization played a role in determining work behavior when unwell [27]. Additionally, nurses’ illness behavior preference, that is, proclivity for presenteeism and perception of illness behavior legitimacy impact work behavior when sick [27]. Head nurses’ internal attitudes, values, and perception preferences are clustered to form the head nurses’ cognitive preference toward presenteeism.

Consequently, we introduced head nurses’ cognitive preference toward presenteeism as a latent and internal individual tendency. Cognition refers to an individual’s knowledge and perception of specific events, including the anticipation of possible events, interpretation of current events, and evaluation of past events [23]. Using this definition, we introduced the head nurses’ cognitive preference towards presenteeism, which implies head nurses’ attitude preference towards presenteeism, including the anticipation of possible presenteeism, the interpretation of current presenteeism, and the evaluation of past presenteeism. Furthermore, anticipation, benefit, and management preferences were examined to examine the individual cognitive preference towards presenteeism. Specifically, anticipation preference refers to an individual behavioral expectation of whether to choose presenteeism while ill, that is, predictions about the occurrence of presenteeism. Benefit preference represents the reasons and consequences of presenteeism, specifically the individual explanatory tendency. Management preference is the negative or positive individual attitude toward their own and others’ presenteeism. As for head nurses, management preference, which includes support and encouragement or objection and intervention, is a distinct behavior of head nurses when subordinate presenteeism exists. Considering the introductory definition of head nurses’ cognitive preference toward presenteeism, the second objective of this study was proposed:

Objective 2: investigate head nurses’ cognitive preference toward presenteeism.

### Head nurses’ cognitive preference toward presenteeism and the relationship with their presenteeism

Head nurses’ cognitive preference towards presenteeism likely impacts their presenteeism. Individual cognitive processing refers to the process through which the brain receives external information and transforms it into internal psychological activities after brain machining, which determines behavior [22]. Therefore, cognition is an important factor influencing behavior, suggesting that various cognitive preferences result in different choices and presentations of behavior. It seems likely that head nurses with different cognitive preferences toward presenteeism would have different choices in terms of sick absences or presenteeism. In particular, more presenteeism would be performed by head nurses who anticipate presenteeism, consider presenteeism a beneficial behavior, and provide positive feedback to subordinates. Existing research has explored supervisors’ attitude preference influence on followers’ presenteeism [14, 28], but has ignored within-individual relationship, that is, that supervisors’ cognitive preference impacts their personal presenteeism. Therefore, the third objective of this study was proposed:

Objective 3: examine the relationship between head nurses’ cognitive preference toward presenteeism and presenteeism among head nurses.

The current research investigated the incidence of presenteeism among head nurses by investigating their cognitive preference via a scenario questionnaire, thereby clarifying the association between head nurses’ cognitive preference toward presenteeism and presenteeism. This study aimed to comprehensively explore the occurrence mechanism of head nurses’ presenteeism and provide protective and intervention strategies for nurses’ presenteeism.

## Methods

### Procedure and participants

This preliminary investigation was a cross-sectional study conducted from July to August 2022, adhering to STROBE guidelines. Prior to investigation, G-power was used to pre-estimate appropriate sample size and has shown that 128 participants is sufficient for examination of the relationship between head nurses’ cognitive preference and presenteeism, with effect size d at 0.5, ɑ err prob at 0.05, and power (1-β err prob) at 0.8 [29]. The participants were recruited from six hospitals located in Zhengzhou, Henan Province, China, through convenience sampling. This study included six provincial public hospitals consisting of three comprehensive hospitals and three specialized hospitals—a cancer hospital, chest hospital, and cardiovascular hospital. Every hospital contains 50–80 departments and approximately 280 departments in total. Excluding sterile and infectious departments, 256 head nurses from 256 departments were recruited to decrease selection bias. Head nurses were eligible if they met the following criteria: (1) agreed to participate in the study, and (2) were front-line head nurses. The exclusion criteria were as follows: (1) worked as head nurses less than six months, and (2) were substitute head nurses.

Prior to the investigation, we communicated with the hospital’s nursing management and obtained their permission. To ensure high quality of collected data, two trained research assistants visited the six hospitals to distribute digital and paper questionnaires that had already been coded. Before completing the questionnaires, all participants were informed of the purpose of the study and provided oral informed consent. The survey was conducted anonymously. Ultimately, the participants returned the completed questionnaires to the research assistants, who performed a simple check to ensure data quality. The Ethical Review Board of the Institution of Psychology and Behavior, Henan University, approved the study design.

### Measures

General demographic characteristics including gender, age, tenure, marital status, educational level, and technical titles were collected. Data on age and tenure were collected and analyzed as the continuous variables. Other information was collected using multiple choice tests. Gender was categorized into male and female, and marital status consisted of unmarried, married, and other. Education level comprised senior high school or technical secondary school, junior college, bachelor’s degree, and master’s degree and above. Technical titles were divided into five categories: nurses, nurse practitioners, nurse-in-charge, deputy chief nurses, and chief nurses.

The Nurse Presenteeism Questionnaire (NPQ) developed by Shan et al. [30] was used to assess the occurrence of presenteeism among head nurses. NPQ comprises 11 items, such as, “Although you felt dizzy or had a headache, you still persevered in going to work.” Participants were asked to recall the number of experiences of attending work in the past six months despite having specific symptoms of illnesses. Each item was rated on a 4-point scale (0 = never, 1 = once, 2 = 2–5 times, 3 = more than 5 times) without reverse scoring. Higher scores represented frequent incidences of presenteeism. The Cronbach’s α of the NPQ in the current study was 0.94.

### Cognitive preference questionnaire

The questionnaire formulated a relatively real scenario in written form [31], specifically, “Xiaowang is a nurse in the inpatient department of a provincial hospital. When she woke up today, she had a cough, runny nose, and slight fever (body temperature is 37.9℃). Considering her health literacy, Xiaowang intends to call the head nurses to inform them about the situation and apply for home rest. At the same time, Xiaowang recognizes that there have been numerous patients in the department recently with a relatively tight workforce and high workload. If she chooses to take a sick absence, it will significantly increase the pressure on all department workers.” The questionnaire consists of six items; one item for anticipation preference (e.g., If you were Xiaowang, you would choose: (1) attend work while ill, (2) rest at home.); four items for benefit preference (e.g., If Xiaowang attended work while ill, from the current/long-term perspective: in your view, Xiaowang’s behavior toward Xiaowang/organization is: (1) beneficial, (2) detrimental.); and one item for management preference (e.g., From the supervisor’s perspective, if Xiaowang attends work while ill, you will: (1) praise her, (2) attention silently, (3) advise her to rest at home). In the pre-study, based on expert evaluation method, five psychological experts and five nursing professionals were invited to individually evaluate the content validity of the scenario. Additionally, all experts were asked to assess Xiaowang’s behavior and whether it could be defined as presenteeism using dichotomous scores. As a result, all experts regarded Xiaowang’s behavior as presenteeism.

### Statistical analysis

The software Statistical Package for the Social Sciences (SPSS version 22.0) was used to analyze the data. Presenteeism in head nurses and head nurses’ cognitive preferences were described using absolute and relative frequencies. An independent-samples t-test was used to evaluate presenteeism against cognitive preference with a 95% confidence interval. Specifically, we randomly selected appropriate samples for data analysis when comparing presenteeism among head nurses with varying cognitive preferences.

## Results

### Demographic characteristics of head nurses

After data cleaning, 233 questionnaires were included in the analysis, with a response rate of 87.9%. Specifically, 12 head nurses could not complete the questionnaire due to emergency work; eight were excluded as they had served as head nurses less than six months; three substitute head nurses were excluded. The survey respondents consisted of 229 females (98.3%) and four males (1.7%). The head nurses aged 30–59 years, with an average age of 42.19 (*SD* = 5.76). The tenure of head nurses ranged from 10 to 41 years, with an average tenure of 22.06 (*SD* = 6.77). Among the participants, 226 (97.0%) were married, four (1.7%) were unmarried, and three (1.3%) responded with “other.” Additionally, 214 (91.8%) had a bachelor’s degree, and 19 (8.2%) had a master’s degree or higher. Regarding technical titles, 126 (54%) had the title of nurse-in-charge or lower, and 107 (45.9%) had the title of deputy chief nurse or higher.

### Common method bias

The results of the common method bias test showed that compared to the original two-factor model (CFI = 0.901, TLI = 0.886, RMSEA = 0.082, SRMR = 0.067), the CFI and TLI of the ULMC model with the added method factor were 0.910 and 0.879, respectively, representing an increase of 0.009 (< 0.1) and 0.007 (< 0.1), respectively. Furthermore, the SRMR and RMSEA of the ULMC with the added method factor were 0.047 and 0.085, respectively, representing a reduction of 0.02 (< 0.05) and 0.003 (< 0.05), respectively. Therefore, serious common method bias was not observed in this study [32].

### Head nurses’ presenteeism and differences in demographic characteristics

According to the head nurses responses, the mean (SD) score for head nurses’ presenteeism was 1.28 (0.82). In the past six months, 225 (96.6%) head nurses had experienced presenteeism at least once. The prevalence of presenteeism among the head nurses is shown in Table 1.

**Table 1:** The incidence of presenteeism among head nurses

Item	M ± SD	Never	Once	2–5 times	More than 5 times	Accumulative incidence
Although you felt dizzy or had a headache, you still persevered in going to work.	1.51 ± 1.00	19.3%	28.8%	33.9%	18.0%	80.7%
Although you felt discomfort in the lower back, you still persevered in going to work	1.76 ± 1.08	15.9%	24.9%	26.6%	32.6%	84.1%
Although you felt chest distress, shortness of breath, or palpitations, you still persevere in going to work.	0.96 ± 1.01	42.9%	28.3%	18.9%	9.9%	57.1%
Although you felt pain or swelling in limbs (and joints), you still persevered in going to work.	1.44 ± 1.17	30.0%	21.9%	22.3%	25.8%	70.0%
Although you felt abdominal pain (including menstrual pain), you still persevered in going to work.	1.58 ± 1.13	23.2%	24.5%	24.0%	28.3%	76.8%
Although you had an upset stomach (e.g., stomachache, flatulence), you still persevered in going to work.	1.24 ± 1.09	32.2%	29.6%	20.6%	17.6%	67.8%
Although you felt whole body fatigue or discomfort, you still persevered in going to work.	1.40 ± 1.09	26.6%	27.0%	26.2%	20.2%	73.8%
Although you experienced nausea and felt like vomiting, you still persevered in going to work.	0.70 ± 0.99	58.8%	21.5%	10.3%	9.4%	41.2%
Although you had a cold (e.g., stuffy nose or cough), you still persevered in going to work.	1.36 ± 1.01	22.7%	35.2%	25.3%	16.7%	77.3%
Although you had a fever, you still persevered in going to work.	0.63 ± 0.91	59.7%	24.0%	9.9%	6.4%	40.3%
Although you had other physical symptoms, you still persevered in going to work.	1.54 ± 1.09	21.5%	28.3%	25.3%	24.9%	78.5%

Based on the cumulative incidence, the main symptoms resulting in presenteeism among head nurses were discomfort in the lower back, dizziness or headache, cold (e.g., stuffy nose or cough), abdominal pain (including menstrual pain), and whole-body fatigue or discomfort.

Additionally, the differences in the head nurses’ NPQ scores according to demographic characteristics were non-significant.

### Head nurses’ cognitive preference toward presenteeism

Table 2 demonstrates the head nurses’ cognitive preferences for presenteeism. In particular, for the response pertaining to management preference, “give praise” and “attention silently” were combined to “give encouragement,” as these responses essentially represent support for presenteeism. Furthermore, “advise her to rest at home” was defined as “conduct discouragement” for brevity.

**Table 2:** Head nurses’ cognitive preference towards presenteeism

Item	Category	Number	Proportion
Anticipation preference	Attend work	10	4.3%
	Rest at home	223	95.7%
Short-term organizational benefit preference	Beneficial	44	18.9%
	Detrimental	189	81.1%
Short-term individual benefit preference	Beneficial	9	3.9%
	Detrimental	224	96.1%
Long-term organizational benefit preference	Beneficial	22	9.4%
	Detrimental	211	90.6%
Long-term individual benefit preference	Beneficial	16	6.9%
	Detrimental	217	93.1%
Management preference	Give encouragement	84	36.1%
	Conduct discouragement	149	63.9%

### Relationship between head nurses’ cognitive preferences and presenteeism

To explore the intrinsic relationship between head nurses’ cognitive preference towards presenteeism and presenteeism among head nurses, an independent-samples t-test was performed. Considering the clearly varying distributions of participants who selected dichotomous options for the items, a matching method was used to reduce analysis deviation. For instance, for anticipation preference, only 10 head nurses chose “attend work while ill,” while 233 participants selected “rest at home.” Ten of the 233 participants who chose “rest at home” were randomly selected and used for the independent-samples t-test. Table 3 illustrates the results.

**Table 3:** Difference in head nurses’ cognitive preference toward presenteeism

Cognitive preference	Category	Number	M ± SD	t	*p*
Anticipation preference	Attend work	10	19.30 ± 8.25	1.499	0.151
	Rest at home	10	13.20 ± 9.87		
Short-term organizational benefit preference	Beneficial	44	13.59 ± 7.12	0.117	0.907
	Detrimental	44	13.39 ± 9.20		
Short-term individual benefit preference	Beneficial	9	13.78 ± 5.45	1.091	0.291
	Detrimental	9	9.56 ± 10.25		
Long-term organizational benefit preference	Beneficial	22	15.55 ± 9.32	0.162	0.872
	Detrimental	22	15.09 ± 9.27		
Long-term individual benefit preference	Beneficial	16	14.75 ± 9.15	-0.458	0.650
	Detrimental	16	16.25 ± 9.36		
Management preference	Give encouragement	84	16.52 ± 9.22	2.600	0.010*
	Conduct discouragement	84	12.81 ± 9.29		

*p < 0.05, **p < 0.01

The results showed that there was no significant difference in presenteeism among head nurses with various benefit and anticipation preferences. However, the difference in presenteeism among head nurses with various management preferences was significant, with *cohen’s d* value at 0.4.

## Discussion

### General discussion

Presenteeism among head nurses is distinct from that among general nurses and affects the work behavior of subordinate nurses and organizational performance. The current study explored the association between presenteeism among head nurses and their cognitive preference for presenteeism based on a preliminary investigation.

The findings demonstrated that 96.6% of head nurses had experienced presenteeism at least once in the past six months, consistent with previous studies [13]. Furthermore, the main symptoms of presenteeism are consistent with a previous study [13] but slight inconsistencies were observed. The incidence of head nurses’ presenteeism were higher than that reported by Shan et al. [14], and the frequency of presenteeism while experiencing fever and discomfort in the lower back were lower and higher, respectively [13]. The high prevalence of presenteeism among head nurses may be due to the exclusive occupational characteristics of a head nurse, especially as caregivers and leaders. Additionally, the time of this investigation may have been significant, resulting in high presenteeism and high frequency of fever. Due to the spread of COVID-19 in 2020, Chinese nurses have been coping with massive workloads and high work-related stress, resulting in more presenteeism,which consistent with previous studies [33]. Additionally, attending work with a fever is rare and risky during the pandemic, as fever is one of the main symptoms of COVID-19. Temperature monitoring and immediate home-based medical observations are recommended, as it reduces the possibility of presenteeism at the institutional and political levels. Finally, participants’ ages may have resulted in a higher frequency of discomfort in the lower back. Participants in this study were older than the nurses recruited by Shan et al. [13]. Based on Lv et al.’s study [34], the prevalence of lower back pain increases with age. Additionally, Hofmann et al. [35] have claimed that the point prevalence of lumbago-sciatica/sciatica in nurses significantly increases with age, and the point prevalence and lifetime prevalence of lower-back pain in nurses slightly increase with age.

Considering the head nurses’ cognitive preferences, the current study divided head nurses’ cognition of presenteeism into anticipation, benefit, and management preferences. Furthermore, we initially investigated head nurses’ cognition of presenteeism using a scenario questionnaire. According to the results, a small minority of head nurses were inclined toward attending work while ill (4.3%) instead of resting at home (95.7%). Head nurses’ anticipation preference for presenteeism is internally associated with their benefit preference; specifically, from both short and long-term perspectives, a vast majority of head nurses are prone to consider presenteeism as a detrimental rather than beneficial behavior for individuals and organizations. Moreover, considering the short- and long-term viewpoints, the percentage of head nurses who considered presenteeism a detrimental behavior was higher than those who regarded it as a beneficial behavior. Interestingly, referring to management preference, 36.1% of 233 head nurses prefer to “give encouragement” to subordinate nurses, which is considerably more than the proportion of head nurses who prefer to attend work while ill (4.3%) and consider presenteeism a beneficial behavior (18.9%; 3.9%; 9.4%; 6.9%). Overall, the results for most head nurses’ anticipation preferences are consistent with their benefit preferences, while being obviously different from their management preferences. The role identity of head nurses generated variance in representing various preferences. The occupational role of head nurses is a combination of caregiver and leader. Head nurses presented their anticipation preference from individual perspectives, indicating subjective opinions. As for benefit preference, head nurses considered it from the perspective of a spectator and evaluated the consequences of others’ presenteeism. Therefore, head nurses’ anticipation and benefit preferences are more likely to be related to their identity as a caregiver, which strengthened their benefit preference that presenteeism is a harmful behavior for individuals. In contrast, to explore management preference, participants were explicitly asked to reflect on their management strategy for subordinate nurses’ presenteeism from a leader’s perspective. When expressing management preference as a leader, the role-identity of leaders is dominant, and the benefit preference is enhanced such that presenteeism is considered a beneficial behavior for the organization. Head nurses’ benefit preference indicates the existence of a difference between the roles of a caregiver and leader in the comprehension of presenteeism, which implies that there is an intra-role conflict among head nurses [36, 37]. This conflict between caregiver and leader further results in variance between the percentage of head nurses with a latently positive attitude toward presenteeism in their anticipation and management preferences.

The current study examined the relationship between head nurses’ cognitive preference toward presenteeism and their presenteeism, and found cognition-behavior inconsistency between anticipation preference, benefit preference, and presenteeism. However, cognition-behavior consistency was observed between management preference and presenteeism. In total, 90% of the head nurses considered presenteeism detrimental to both individuals and organizations and tended to rest at home while ill. However, the NPQ results showed that more than 90% of the head nurses had experienced presenteeism in the past six months. These findings exhibit a distinct contradiction between head nurses’ presenteeism and their anticipation and benefit preferences. This cognition-behavior inconsistency indicates that head nurses’ behavioral decisions, including choosing to attend work while ill and taking sick absences, are not only related to cognitive factors but also based on the comprehensive evaluations of various factors. Previous studies have claimed that work irreplaceability [38, 39] and high job demands [40] are essential factors influencing the high incidence of presenteeism among head nurses. Furthermore, Ingwell-Spolan [15] has argued that head nurses are in a demanding, multifaceted, and complicated position that requires high levels of accountability. Therefore, they are prone to attending work while being unwell. Nonetheless, there is consistency between head nurses’ presenteeism and management preferences. Specifically, head nurses who tend to “give encouragement” have significantly higher NPQ scores than those inclined toward “conduct discouragement.” It is known that leaders’ behavioral integrity has positive impacts on supervisors and organizations, such as improving followers’ loyalty to supervisors and further increasing followers’ loyalty to organizations [41]. It is likely that a hypocritical leader with word-deed misalignment will face difficulty in repairing their reputation; their followers will feel worthless, unhappy, indignant, and restless, and the followers’ motivation, job satisfaction, organization commitment, and performance will also be negatively affected. Further, the organization will favor the formation of significant interpersonal relationships, resulting in an increase in organizational gossip as well as a tense and unreliable work environment [42, 43]. To establish a positive image, head nurses maintain leadership authority, promote subordinate loyalty and increase management efficiency. Therefore, head nurses who were inclined to “give encouragement” to followers’ presenteeism showed higher presenteeism scores.

### Implications

This study makes theoretical and empirical contributions. First, emphasizing the influence of cognitive factors on presenteeism, we introduced cognitive preference toward presenteeism, and divided the factor into anticipation preference, benefit preference, and management preference, which advance our understanding of the boundary conditions of presenteeism. Second, the Cognitive Preference Questionnaire was developed based on experimental vignette methodology to assess head nurses’ cognitive preference toward presenteeism, thus providing tools for future research and enriching our knowledge of presenteeism. Third, from the perspective of role identity, we focused on the occupational characteristics of head nurses as both caregivers and leaders and discussed the inconsistency between the expectation, benefit, and management preferences of head nurses according to their dual roles. The findings not only provide theoretical support for introducing cognitive preference in the research on presenteeism but also expand Rainbow and Steege’s [5] theoretical model of presenteeism among nurses. Third, the present study illuminates the high incidence of presenteeism among head nurses, which is remarkably higher than that among general nurses. Combining the practical incidence of presenteeism among head nurses and their occupational characteristics as head nurses, interventions for preventing nurses’ presenteeism should focus on head nurses, considering it as a starting point to create a favorable occupational climate. Additionally, the consistency between head nurses’ management preference toward presenteeism and their presenteeism confirms that cognition is an important factor affecting presenteeism. Therefore, cognitive factors should be emphasized in presenteeism interventions aimed at nurses. More specifically, the nursing workforce should be guided to recognize presenteeism and make rational choices when feeling unwell. Furthermore, the consistency between head nurses’ anticipation and benefit preferences for presenteeism and their presenteeism implies that in addition to cognitive factors, various other factors affect individual decision-making when ill. Consequently, to develop an effective intervention for preventing nurses’ presenteeism, researchers should not only focus on cognitive factors at the individual level but also target the effects of personal emotion [44], personality [45], family-job relationship [46], job demands [47], and job resources [46]. Moreover, various determinants from the individual, team, and organizational levels should be considered to create comprehensive, integral, and global interventions.

### Limitations

Although the current study expands the current literature on presenteeism, it has some limitations. First, this study was conducted during the COVID-19 pandemic, and the results showed a high incidence of head nurses’ presenteeism. The high prevalence can be explained by the dual-role (caregiver and leader) of head nurses and the increased workload of nurses during the pandemic period, warranting further examination. This resulted in uncertainty regarding the generalizability of the present findings to ordinary situations. Additionally, this study used an original scenario questionnaire to measure head nurses’ cognitive preferences. Although relevant psychology and nursing experts were invited to evaluate the validity of the questionnaire during its development, more studies are needed to retest the questionnaire’s validity and applicability. Third, participants in this study were recruited from six public hospitals in Zhengzhou, Henan Province, China, through convenience sampling. Although we recruited all the head nurses in available departments, selection bias was inevitable. Future studies should collect data from nurses in different regions to verify the findings of this study.

## Conclusion

Existing research has confirmed a high prevalence of presenteeism among nurses, which leads to multifaceted negative consequences. This current research focused on the uniqueness and importance of head nurses in the nursing department and investigated the incidence of presenteeism among them. Furthermore, the current study comprehensively examined the occurrence mechanism of head nurses’ presenteeism by creatively introducing cognitive preference and initially exploring the association between head nurses’ cognitive preference towards presenteeism and their presenteeism. The results indicated a significantly high incidence of presenteeism among head nurses; although, most of them denied having a cognitive preference towards presenteeism. Both cognition-behavior consistency and inconsistency were demonstrated in the relationship between various cognitive preferences and head nurses’ presenteeism. Our findings suggest that presenteeism interventions aimed at nurses should focus on head nurses and their cognitive factors. In addition to cognitive factors, extra individual and organizational factors should also be considered to establish an effective intervention.

## Data Availability

The datasets produced for this current study are available upon request from the corresponding author.
